# The effect of combining manual therapy with exercise for mild chronic obstructive pulmonary disease: study protocol for a randomised controlled trial

**DOI:** 10.1186/s13063-017-2027-z

**Published:** 2017-06-17

**Authors:** Roger M. Engel, Jaxson Wearing, Peter Gonski, Subramanyam Vemulpad

**Affiliations:** 1 Department of Chiropractic, Macquarie University, North Ryde, Sydney, NSW 2109 Australia; 2 Southcare, Sutherland Hospital, Sydney, Australia

**Keywords:** Chronic obstructive pulmonary disease, COPD, Manual therapy, Spinal manipulative therapy, Pulmonary rehabilitation, Exercise, Randomised controlled trial, Lung function, Trial protocol

## Abstract

**Background:**

Chronic obstructive pulmonary disease (COPD) is a major cause of disability and hospital admission. Current management strategies have not been successful in altering the loss of lung function typically seen as the disease progresses. A recent systematic review into the use of spinal manipulative therapy (SMT) in the management of COPD concluded that there was low level evidence to support the view that a combination of SMT and exercise had the potential to improve lung function more than exercise alone in people with moderate to severe COPD.

The aim of this study is to investigate whether the combination of exercise and manual therapy (MT) that includes SMT produces sustainable improvements in lung function and exercise capacity in people with mild COPD.

**Methods/design:**

The study is a randomised controlled trial of 202 people with stable mild COPD. The cohort will be divided into two equal groups matched at baseline. The first group will receive a standardised exercise program. The second group will receive MT that includes SMT plus the same standardised exercise program. Exercise will be administered a total of 36 times over an 18-week period, while MT will be administered in conjunction with exercise a total of 15 times over a 6-week period. The primary outcome measure is lung function (forced expiratory volume in the 1^st^ second: FEV_1_ and forced vital capacity: FVC). The secondary outcome measures are the 6-minute walking test (6MWT), quality of life questionnaire (St George’s Respiratory Questionnaire: SGRQ), anxiety and depression levels (Hospital Anxiety and Depression Scale: HADS), frequency of exacerbations, chest wall expansion measurements (tape measurements) and systemic inflammatory biomarker levels. Outcome measurements will be taken by blinded assessors on seven occasions over a 48-week period. Adverse event data will also be gathered at the beginning of each intervention session.

**Discussion:**

This randomised controlled trial is designed to investigate whether the combination of MT and exercise delivers any additional benefits to people with mild COPD compared to exercise alone. The study is designed in response to recommendations from a recent systematic review calling for more research into the effect of MT in the management of COPD.

**Trial registration:**

ANZCTRN, 12614000766617. Registered on 18 July 2014.

**Electronic supplementary material:**

The online version of this article (doi:10.1186/s13063-017-2027-z) contains supplementary material, which is available to authorized users.

## Background

Chronic obstructive pulmonary disease (COPD) is a major cause of chronic morbidity and mortality. It is responsible for a substantial and increasing proportion of the economic and social burden of disease and is currently ranked as the fourth leading cause of death worldwide [1, 2]. The disease is characterised by progressive loss of lung function and includes symptoms such as dyspnea, sputum production and cough [1, 2]. While lung function tests are the primary method used to diagnose and track disease progression, COPD is associated with other comorbidities such as depression, cardiovascular disease and skeletal muscle dysfunction which also influence progression [1].

As exercise capacity is a prognostic indicator in COPD, any skeletal muscle dysfunction that reduces exercise performance can affect disease severity over time by reducing exercise capacity [2]. In COPD, the primary source of exercise limitation is dyspnea, which curtails exercise performance before maximum capacity has been reached. Exercise performance has therefore become a target for therapeutic intervention with both international and Australian guidelines now including exercise training as a standard component in pulmonary rehabilitation [1, 2]. One of the causes of exercise-limiting dyspnea is altered chest wall mechanics which includes an increase in chest wall rigidity (CWR) [3–6]. This increase results in a fall in the efficiency of the ventilatory pumping mechanism and an increase in the effort required to breathe [5, 7–10]. Addressing the increase in CWR has been suggested as a way of reducing or delaying the onset of exercise-limiting dyspnea [11].

The increase in CWR typically seen in COPD is initially the result of expansionary forces exerted on the chest wall by the development of lung hyper-inflation [12]. Over time, this increase is perpetuated by the mechanical properties of the respiratory muscles which resist changes in length through a process known as thixotropy [13, 14]. The thixotropic properties of a muscle are determined by the position that muscle is held in just prior to contraction. In the case of the respiratory muscles, lung hyper-inflation places these muscles in either a shortened or lengthened position prior to contraction. Altering the resting length of these muscles would reduce CWR and therefore mitigate one of the factors responsible for producing exercise-limiting dyspnea.

While pulmonary rehabilitation (PR) has been shown to increase exercise capacity, it has not been shown to be capable of delivering any clinically meaningful increases in lung function [15–17]. Administering an intervention capable of reducing CWR in conjunction with exercise has the potential of producing an increase in exercise capacity and lung function through a synergy between interventions.

Manual therapy (MT) is an intervention capable of producing a short-term reduction in CWR in people with COPD. A recent systematic review which investigated the effect of spinal manipulative therapy (SMT), a form of MT, in the management of COPD recommended the need for further research into the use of SMT with exercise for people with COPD [18]. While acknowledging that the studies reviewed used small sample sizes, the reviewers concluded that the quality of the evidence showing that the combination of SMT and exercise had the potential to produce clinically meaningful improvements in lung function and exercise capacity compared to exercise alone was high [18]. This finding had not previously been reported in the literature and provides an opportunity to improve the outcomes of PR [19, 20].

The primary aim of this trial is to evaluate the effect on lung function of administering MT that includes SMT in conjunction with exercise to people with mild COPD. The secondary aim is to investigate the effect of this combination of interventions on exercise capacity, quality of life, anxiety and depression and systemic inflammatory biomarkers.

## Methods/design

### Study design

The trial is designed as a randomised controlled trial and fulfils the requirements of the Standard Protocol Items: Recommendations for Interventional Trials (SPIRIT) checklist [21] (Additional file 1). It will evaluate 202 participants randomly allocated to two equal groups. Participants in Group 1 (Ex) will undergo an exercise regime that has been modelled on an existing pulmonary rehabilitation program; participants in Group 2 (SM + Ex) will have MT which includes SMT administered to their thoracic spine and ribs just prior to performing the same exercise regime as Group 1. Participants will be recruited through direct referral from their respiratory specialist or general practitioner or through self-referral in response to public advertisements calling for volunteers for the trial. The trial is being conducted in the pulmonary rehabilitation department at Sutherland Hospital in Sydney, Australia.

### Participant characteristics

The trial includes both males and females between the ages of 50 and 65 years of age with a forced expiratory volume in the 1^st^ second (FEV_1_)/forced vital capacity (FVC) ratio of < 0.7 and an FEV_1_% predicted of 60–80% (COPDX 2016: mild COPD). Participants will be excluded if they are currently smoking, cannot complete a 6-minute walking test (6MWT) unassisted, have a history of autoimmune disease or are contra-indicated to SMT. Inclusion and exclusion criteria are described in Table 1.

**Table 1:** Inclusion and exclusion criteria for participation

Inclusion criteria	Exclusion criteria
• Male and female • 50–65 years • Mild COPD (60% ≤ FEV_1_% < 80%: COPDX) • Stable COPD (no exacerbations in preceding 6 months) • Non-smoking (for preceding 6 months) • Willingness to provide written consent • Willingness to participate in and comply with the study requirements	• Inability to complete 6-minute walking test unassisted • History of auto-immune disease, e.g. RA or SLE that may have altered systemic inflammatory biomarkers • Contra-indicated to thoracic spinal manipulation o Bone density (DEXA) scores below minimum levels (T score < –2.5 and/or Z score < –1) o Thoracic joint instability o Acute pain on thoracic joint range of motion testing o Advanced chest wall muscle wasting o High level of anxiety related to receiving thoracic spinal manipulation • Inability to understand English • Inability to provide informed consent, e.g. people with a cognitive impairment, an intellectual disability or a mental illness • Completed a PR program in the previous 12 months

*COPD* chronic obstructive pulmonary disease, *COPDX* the COPD-X Plan. Australian and New Zealand guidelines for the management of chronic obstructive pulmonary disease. Version 2.45. March 2016, *FEV*_*1*_ *%* forced expiratory volume in the 1^st^ second percent predicted (age-matched), *RA* rheumatoid arthritis, *SLE* systemic lupus erythematosus, *DEXA* dual energy X-ray absorptiometry, *PR* pulmonary rehabilitation

### Protocol description

**Fig. 1:**
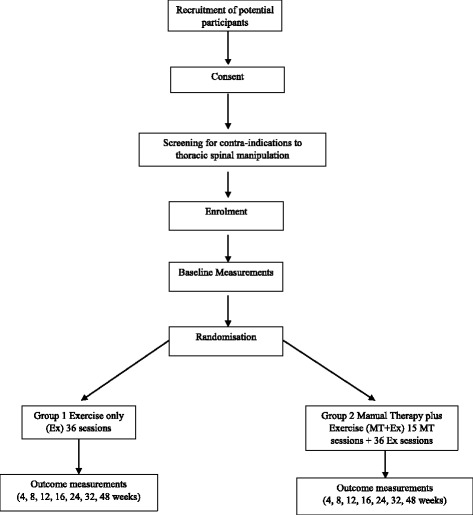
Flow of participants through the trial. *MT* manual therapy, *Ex* exercise

Volunteers who meet the inclusion criteria will be given an information sheet and asked to provide written consent to participate in the trial. They will then undergo a physical screening examination for the presence of contra-indications to thoracic MT. After successfully passing this screening, they will be enrolled in the trial, given a trial-specific identification (ID) number and allocated to a group sequentially using a computer-generated random sequence list drawn up prior to the start of the trial by a member of the staff not involved in any other aspect of the trial. Baseline measurements for each participant are then taken. These include lung function assessment using spirometry, exercise capacity (6MWT), chest wall expansion (tape measure), quality of life (St George’s Respiratory Questionnaire: SGRQ), anxiety and depression levels (Hospital Anxiety and Depression Scale: HADS) and systemic inflammatory biomarker levels via blood sampling (C-reactive protein (CRP) and leukocyte count). The study protocol flow of participants is outlined in Fig. 1.

**Fig. 2:**
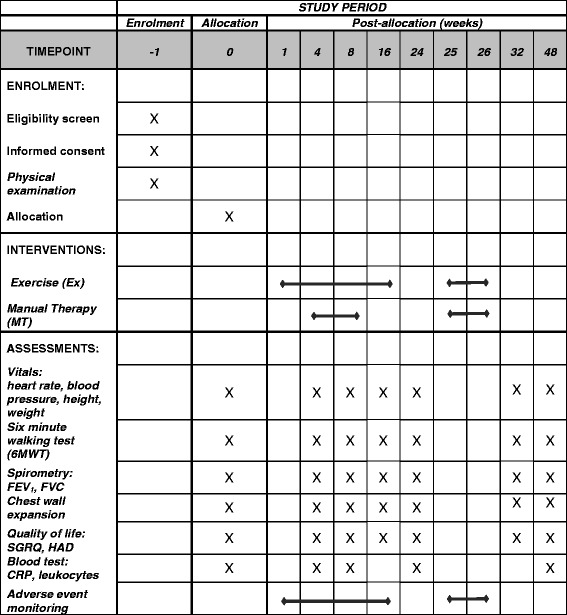
SPIRIT figure showing time points for enrolment, interventions and assessments. *FEV*_*1*_ Forced expiratory volume in the 1^st^ second, *FVC* Forced vital capacity, *SGRQ* St George’s Respiratory Questionnaire, *HAD* Hospital Anxiety and Depression (scale), *CRP* C-reactive protein

All outcome measures will be taken by staff of the hospital familiar with administering these assessments and blinded to a participant’s group allocation. Lung function measurements are measured in the sitting position and include FEV_1_ and FVC. Exercise capacity is assessed using the 6MWT where capacity is determined by the total distance walked on a flat surface (hospital hallway) in a period of 6 minutes. Quality of life (SGRQ) and anxiety and depression (HADS) scores are calculated using the standard procedures for these questionnaires. Frequency of exacerbations is gathered by direct questioning of a participant. Systemic inflammatory biomarkers are measured via blood sampling by the hospital’s pathology department from samples (10 mL) collected by a registered nurse who is blinded to a participant’s group allocation. The SPIRIT figure showing the respective time points for enrolment, interventions and assessments is provided in Fig. 2.

All MT is administered by chiropractors and/or osteopaths with more than 5 years of experience in the manual therapy protocol (MTP) used in this trial. This is the same MTP that was used in two previous trials on patients with COPD [22, 23]. It consists of a combination of soft tissue (ST) therapy and thoracic spinal manipulation (SM).

The ST component of the MTP consists of gentle effleurage and cross-fibre friction therapy applied to the muscles of the posterior chest wall including the intercostal, serratus posterior and anterior, rhomboid, trapezius, latissimus dorsi, erector spinae, quadratus lumborum and levator scapulae muscles. The SM component consists of two separate manipulations (Grade V mobilisation [24]). Each manipulation involves the delivery of a high-velocity low-amplitude (HVLA) posterior-to-anterior force directed towards the intervertebral, costovertebral and costotransverse joints. The first manipulation is administered at the level of the upper/middle thoracic spine, while the second is administered at the level of the middle/lower thoracic spine. All SM are administered as non-specific, multi-joint (group) manipulations. Administering SM in this way reduces the total number of manipulations required in a single session, as each manipulation has the potential to affect several thoracic vertebrae and their associated ribs simultaneously. An MT session lasts approximately 20 minutes and is administered just prior to exercise intervention in the MT + Ex Group.

### Consent process

The consent process followed in this trial will be the same for all participants. Potential participants will receive the Patient Information and Consent Form (PICF) explaining the trial’s processes and procedures, including consent to publish, and what is expected from them during the trial. Each participant will be given the opportunity to ask questions about their involvement in the trial and to have those questions answered by a researcher associated with the trial prior to providing consent. A trial-specific ID number will be used when gathering and recording all information about a participant. A list containing the contact details of all participants and their corresponding ID numbers will be kept separate from all other data. All paper copy forms will be stored in a locked filing cabinet in the hospital facility during the trial. Once the intervention phase of the trial has been completed (week 26), all data from these files will be transferred to a password-protected encrypted file and stored on the chief investigator’s university computer located in his locked university office. These files will be backed up on an external hard drive and stored in a locked filing cabinet in the university office of another investigator. The trial was approved by the South Eastern Sydney Local Health District - Human Research Ethics Committee (HREC): approval number 13/004.

### Sample size calculation

The sample size calculation was based on FVC. The minimum clinically important difference for FVC is 200 mL with the standard deviation obtained from previous studies [22, 23]. With a power of 0.8 (80%) and an alpha of 0.05, the minimum sample size per group is 92. Assuming a drop-out rate of 10%, the minimum cohort size would be 202 (two groups of 101).

### Statistical analysis

Data will be reported as group means, standard deviations and 95% confidence intervals. Analysis will be performed as an analysis of covariance (ANCOVA) for differences between groups with baseline as a covariate. Standard errors will be calculated using a non-parametric bootstrap to allow for the different error variances for each group. A *p* value of < 0.05 has been set for statistical significance. For outcomes found to be statistically significant, the proportion of participants with a change greater than the minimum clinically important difference will be calculated for each outcome. The number needed to treat will be calculated using Bender’s method for confidence intervals. Missing data will be accounted for by using an intention-to-treat analysis with data from subjects lost to follow-up imputed using the multiple imputation method.

Two interim analyses are planned before the final analysis. They are mid-trial following the second blood test (week 24) and 6 weeks after completion of all interventions (week 32). The conduct of the trial will not be affected by the results of these interim analyses.

### Data monitoring

A Data Safety Monitoring Board (DSMB) has been appointed to oversee the study. It consists of a respiratory physician, a unit nurse manager, the hospital’s governance officer and an experienced manual therapist clinician. The DSMB will meet every 4 months or as needed to review the study data.

### Adverse events

The risks of harm or discomfort to participants in this project primarily relate to the potential for adverse events (AEs) resulting from MT intervention. The majority of reported AEs associated with MT are mild (muscle soreness and local discomfort), self-limiting, require no further medical attention and resolve within 48 hours [25]. Moderate AEs such as fracture, have also been reported following SMT and have been estimated to occur at a rate of 1 in 40,000 [26]. Reports of major or catastrophic AEs resulting from spinal manipulation have appeared in the literature with nearly all associated with neck (cervical) manipulation. This trial involves manipulation of the thoracic spine and ribs only and does not include any neck manipulation. The MTP used in this trial is the same protocol as the one used in two previous trials on people with COPD [22, 23]. There were no major or moderate AEs reported in either of those trials. Mild AEs associated with MT intervention were reported at a rate of 15% (18 out of 112) in the trial on patients with moderate COPD (average age 56.1 years) [22] and at a rate of less than 1% (2 out of 403) in the trial on patients with moderate to severe COPD (average age 65.5 years) [23].

As the MTP used in the trial being reported here is the same as the one used in the two previous Australian trials, it is reasonable to predict that the risk of harm or discomfort to participants will be at a similar rate to those of the previous trials.

## Discussion

In COPD, disease progression is marked by declining lung function and includes predictors of morbidity and mortality such as exercise capacity and exacerbations. While pulmonary rehabilitation has demonstrated improvements in exercise capacity and quality of life measures, it has yet to show any clinically meaningful improvements in lung function. Administering a combination of MT and exercise has been shown to have the potential to deliver improvements in lung function and exercise capacity by reducing CWR and delaying the onset of exercise-limiting dyspnea, thereby facilitating an increase in exercise performance. We hypothesize that over time this would have a cumulative beneficial effect on exercise capacity.

For this approach to reach its maximum potential, the combination of interventions would need to be administered earlier in the disease cycle (i.e. in the mild COPD stage) before a greater proportion of lung function and exercise capacity has been lost. Administering an intervention that improves the long-term prognosis of COPD through increasing exercise capacity may also help to improve the uptake of exercise as a possible preventative measure against disease progression.

The current Australian guidelines recommend PR for people with all stages of COPD [2]. However, figures show that only 5—10% of Australians with moderate to severe COPD access PR services [27]. While there are a number of reasons for such a low uptake, including poor service availability, compliance has been identified as one of the reasons. Including MT intervention in PR programs may help remedy this if it is shown to enhance the benefits of PR.

The randomised controlled trial being reported in this manuscript is designed to examine the effect of a combination of MT and exercise on lung function and exercise capacity in people with mild COPD. The trial’s design satisfies the recommendations of a recent systematic review into the use of SMT in the management of COPD [18] in that it is adequately powered, configured to have a low risk of bias and has the potential to deliver meaningful data for the use of MT in the management of COPD.

## Trial status

Recruitment for the trial is ongoing at the time of submission.

## Additional file

Additional file 1: SPIRIT 2013 checklist. Recommended items to address in a clinical trial protocol and related documents. (PDF 168 kb)

## Abbreviations

6MWT: 6-Minute walking test
AE: Adverse event
ANCOVA: Analysis of covariates
COPD: Chronic obstructive pulmonary disease
CRP: C-reactive protein
CWR: Chest wall rigidity
DEXA: Dual energy X-ray absorptiometry
DSMB: Data Safety Monitoring Board
Ex: Exercise
FEV_1_: Forced expiratory volume in the 1^st^ second
FVC: Forced vital capacity
HADS: Hospital Anxiety and Depression Scale
HVLA: High-velocity low-amplitude
ID: Identification
MT: Manual therapy
MTP: Manual therapy protocol
PICF: Patient Information and Consent Form
PR: Pulmonary rehabilitation
RA: Rheumatoid arthritis
RCT: Randomised controlled trial
SGRQ: St George’s Respiratory Questionnaire
SLE: Systemic lupus erythematous
SM: Spinal manipulation
SMT: Spinal manipulative therapy
ST: Soft tissue

## References

[1] Global strategy for the diagnosis, management and prevention of COPD 2017 (GOLD). http://goldcopd.org/gold-2017-global-strategy-diagnosis-management-prevention-copd/. Accessed 15 June 2017.

[2] The COPD-X Plan: Australian and New Zealand guidelines for the management of chronic obstructice pulmonary disease 2017. Version 2.49. http://copdx.org.au/. Accessed 15 June 2017.

[3] Pitta F, Troosters T, Spruit MA, Probst VS, Decramer M, Gosselink R (2005). Characteristics of physical activities in daily life in chronic obstructive pulmonary disease. Am J Respir Crit Care Med.

[4] Theander K, Jakobsson P, Jörgensen N, Unosson M (2009). Effects of pulmonary rehabilitation on fatigue, functional status and health perceptions in patients with chronic obstructive pulmonary disease: a randomized controlled trial. Clin Rehabil.

[5] O’Donnell DE, Ora J, Webb KA, Laveneziana P, Jensen D (2009). Mechanisms of activity-related dyspnea in pulmonary diseases. Respir Physiol Neurobiol.

[6] Pride N, Macklem PT. Lung mechanics in disease. Chapter 1.1: Chronic Obstructive Pulmonary Disease. Compr Physiol. 1986.

[7] O’Donnell DE, Laveneziana P (2007). Dyspnea and activity limitation in COPD: mechanical factors. COPD: J Chron Obstruct Pulmon Dis.

[8] Pepin V, Saey D, Laviolette L, Maltais F (2007). Exercise capacity in chronic obstructive pulmonary disease: mechanisms of limitation. COPD: J Chron Obstruct Pulmon Dis.

[9] Ranieri V, Giuliani R, Mascia L, Grasso S, Petruzzelli V, Bruno F (1996). Chest wall and lung contribution to the elastic properties of the respiratory system in patients with chronic obstructive pulmonary disease. Eur Respir J.

[10] Aliverti A, Kayser B, Macklem PT (2007). A human model of the pathophysiology of chronic obstructive pulmonary disease. Respirology.

[11] Engel R, Vemulpad S (2011). The role of spinal manipulation, soft-tissue therapy, and exercise in chronic obstructive pulmonary disease: a review of the literature and proposal of an anatomical explanation. J Altern Complement Med.

[12] Brashers VL, Davey S (2006). Alterations of pulmonary function. Pathophysiology: the biologic basis for disease in adults and children.

[13] Izumizaki M, Iwase M, Ohshima Y, Homma I (2006). Acute effects of thixotropy conditioning of inspiratory muscles on end-expiratory chest wall and lung volumes in normal humans. J Appl Physiol.

[14] Proske U, Morgan DL, Gregory JE (1993). Thixotropy in skeletal muscle and in muscle spindles: a review. Prog Neurobiol.

[15] Bestall J, Paul E, Garrod R, Garnham R, Jones P, Wedzicha J (2003). Longitudinal trends in exercise capacity and health status after pulmonary rehabilitation in patients with COPD. Respir Med.

[16] Pitta F, Troosters T, Probst VS, Langer D, Decramer M, Gosselink R (2008). Are patients with COPD more active after pulmonary rehabilitation?. Chest J.

[17] Laviolette L, Bourbeau J, Bernard S, Lacasse Y, Pepin V, Breton M-J (2008). Assessing the impact of pulmonary rehabilitation on functional status in COPD. Thorax.

[18] Wearing J, Beaumont S, Forbes D, Brown B, Engel R (2016). The use of spinal manipulative therapy in the management of chronic obstructive pulmonary disease: a systematic review. J Altern Complement Med.

[19] Garcia-Aymerich J, Agustí À, Barberà JA, Belda J, Farrero E, Ferrer A (2009). Phenotypic heterogeneity of chronic obstructive pulmonary disease. Arch Bronconeumol (English Edition).

[20] Celli B, MacNee W, Agusti A, Anzueto A, Berg B, Buist A (2004). Standards for the diagnosis and treatment of patients with COPD: a summary of the ATS/ERS position paper. Eur Respir J.

[21] Chan A-W, Tetzlaff JM, Altman DG, Laupacis A, Gøtzsche PC, Krleža-Jerić K, Hróbjartsson A, Mann H, Dickersin K, Berlin J, Doré C, Parulekar W, Summerskill W, Groves T, Schulz K, Sox H, Rockhold FW, Rennie D, Moher D (2013). SPIRIT 2013 Statement: Defining standard protocol items for clinical trials. Ann Intern Med.

[22] Engel RM, Vemulpad SR, Beath K (2013). Short-term effects of a course of manual therapy and exercise in people with moderate chronic obstructive pulmonary disease: a preliminary clinical trial. J Manip Physiol Ther.

[23] Engel RM, Gonski P, Beath K, Vemulpad S (2014). Medium term effects of including manual therapy in a pulmonary rehabilitation program for chronic obstructive pulmonary disease (COPD): a randomized controlled pilot trial. J Man Manip Ther.

[24] Maitland GD (2014). Maitland’s vertebral manipulation. 8^th^ ed. Eds: Hengeveld E, Banks K, editors.

[25] Carnes D, Mars TS, Mullinger B, Froud R, Underwood M (2010). Adverse events and manual therapy: a systematic review. Man Ther.

[26] Ernst E (2007). Adverse effects of spinal manipulation: a systematic review. J R Soc Med.

[27] Australian Institute of Health & Welfare (AIHW). Monitoring pulmonary rehabilitation and long-term oxygen therapy for people with chronic obstructive pulmonary disease (COPD) in Australia. Cat. no. ACM 29. http://www.aihw.gov.au/publication-detail/?id=60129545161. Accessed 15 June 2017.

